# Factors associated with 90-day mortality in Vietnamese stroke patients: Prospective findings compared with explainable machine learning, multicenter study

**DOI:** 10.1371/journal.pone.0310522

**Published:** 2024-09-20

**Authors:** Ton Duy Mai, Dung Tien Nguyen, Cuong Chi Tran, Hai Quang Duong, Hoa Ngoc Nguyen, Duc Phuc Dang, Hai Bui Hoang, Hong-Khoi Vo, Tho Quang Pham, Hoa Thi Truong, Minh Cong Tran, Phuong Viet Dao

**Affiliations:** 1 Faculty of Stroke and Cerebrovascular Disease, University of Medicine and Pharmacy, Vietnam National University, Ho Chi Minh City, Vietnam; 2 Stroke Center, Bach Mai Hospital, Hanoi, Vietnam; 3 Hanoi Medical University, Hanoi, Vietnam; 4 Stroke International Services (S.I.S) General Hospital, Can Tho City, Vietnam; 5 Da Nang Hospital, Da Nang City, Vietnam; 6 Nghe An Friendship General Hospital, Vinh City, Vietnam; 7 Military Medical Academy, Hanoi, Vietnam; 8 Hanoi Medical University Hospital, Hanoi, Vietnam; 9 Department of Neurology, University of Medicine and Pharmacy, Vietnam National University, Hanoi, Vietnam; 10 Center of Neurology, Hanoi, Vietnam; 11 Nuffield Department of Clinical Neuroscience, University of Oxford, Oxford, United Kingdom; Foshan Sanshui District People’s Hospital, CHINA

## Abstract

The prevalence and predictors of mortality following an ischemic stroke or intracerebral hemorrhage have not been well established among patients in Vietnam. 2885 consecutive diagnosed patients with ischemic stroke and intracerebral hemorrhage at ten stroke centres across Vietnam were involved in this prospective study. Posthoc analyses were performed in 2209 subjects (age was 65.4 ± 13.7 years, with 61.4% being male) to explore the clinical characteristics and prognostic factors associated with 90-day mortality following treatment. An explainable machine learning model using extreme gradient boosting and SHapley Additive exPlanations revealed the correlation between original clinical research and advanced machine learning methods in stroke care. In the 90 days following treatment, the mortality rate for ischemic stroke was 8.2%, while for intracerebral hemorrhage, it was higher at 20.5%. Atrial fibrillation was an elevated risk of 90-day mortality in the ischemic stroke patient (OR 3.09; 95% CI 1.90–5.02, p<0.001). Among patients with intracerebral hemorrhage, there was no statistical significance in those with hypertension compared to their counterparts without hypertension (OR 0.65, 95% CI 0.41–1.03, p > 0.05). The baseline NIHSS score was a significant predictor of 90-day mortality in both patient groups. The machine learning model can predict a 0.91 accuracy prediction of death rate after 90 days. Age and NIHSS score were in the top high risks with other features, such as consciousness, heart rate, and white blood cells. Stroke severity, as measured by the NIHSS, was identified as a predictor of mortality at discharge and the 90-day mark in both patient groups.

## Introduction

Vietnam, a developing country in Southeast Asia, grapples with the challenge of a dual disease burden, encompassing both communicable and non-communicable diseases [1]. Among these conditions, stroke is the world’s biggest killer and is growing in incidence [2]. In recent years, stroke centres and units have been established nationwide to address this concern. Advanced technology supporting stroke and stroke care medicine has been introduced. Although the demand could partially be met by the present number of stroke facilities, an increase in access to stroke units across the country has marked an important milestone in the advancement of stroke care in Vietnam [3].

Based on our current understanding, there appears to be a lack of research data in Vietnam about the comprehensive evaluation of prevalence rates, clinical characteristics, and post-treatment outcomes, specifically within the 90-day timeframe, in consecutive stroke patients receiving care within stroke facilities [4]. Determining factors affecting mortality in stroke patients is crucial for assisting clinicians in identifying and categorizing high-risk patient cohorts. Subsequently, this knowledge facilitates the implementation of tailored intervention strategies to improve patient survival outcomes. Machine learning is a useful tool to identify potential risks, especially in clinical settings. However, there is limited work on machine learning in supporting stroke care in Vietnam.

Therefore, in this paper, we perform a post-hoc analysis to identify risk factors from a prospective study involving 2,285 consecutive patients diagnosed with both ischemic stroke and intracerebral hemorrhage. These data were collected at ten specialized stroke centers across Vietnam. This study identifies factors associated with mortality rate after 90 days of treatment in stroke patients. Clinical statistical analysis will be performed to analyse the mortality risks of post-stroke patients. In parallel, an explainable machine learning model using extreme gradient boosting and the SHapley Additive exPlanations will be applied to the dataset to learn the pattern and rank the importance of features to compare with ordinary clinical results.

## Methodology

### Participants and ethical approval

#### Study approval statement

This prospective observational study was conducted across 10 stroke units, departments, and centres in Vietnam from August 1^st^ to August 31^st^, 2022. The study obtained approval from the ethics committee of Bach Mai Hospital (NO– 1575/QĐ-BVBM). The research process and research objectives were explained to the patient.

#### Consent to participate statement

**Fig 1:**
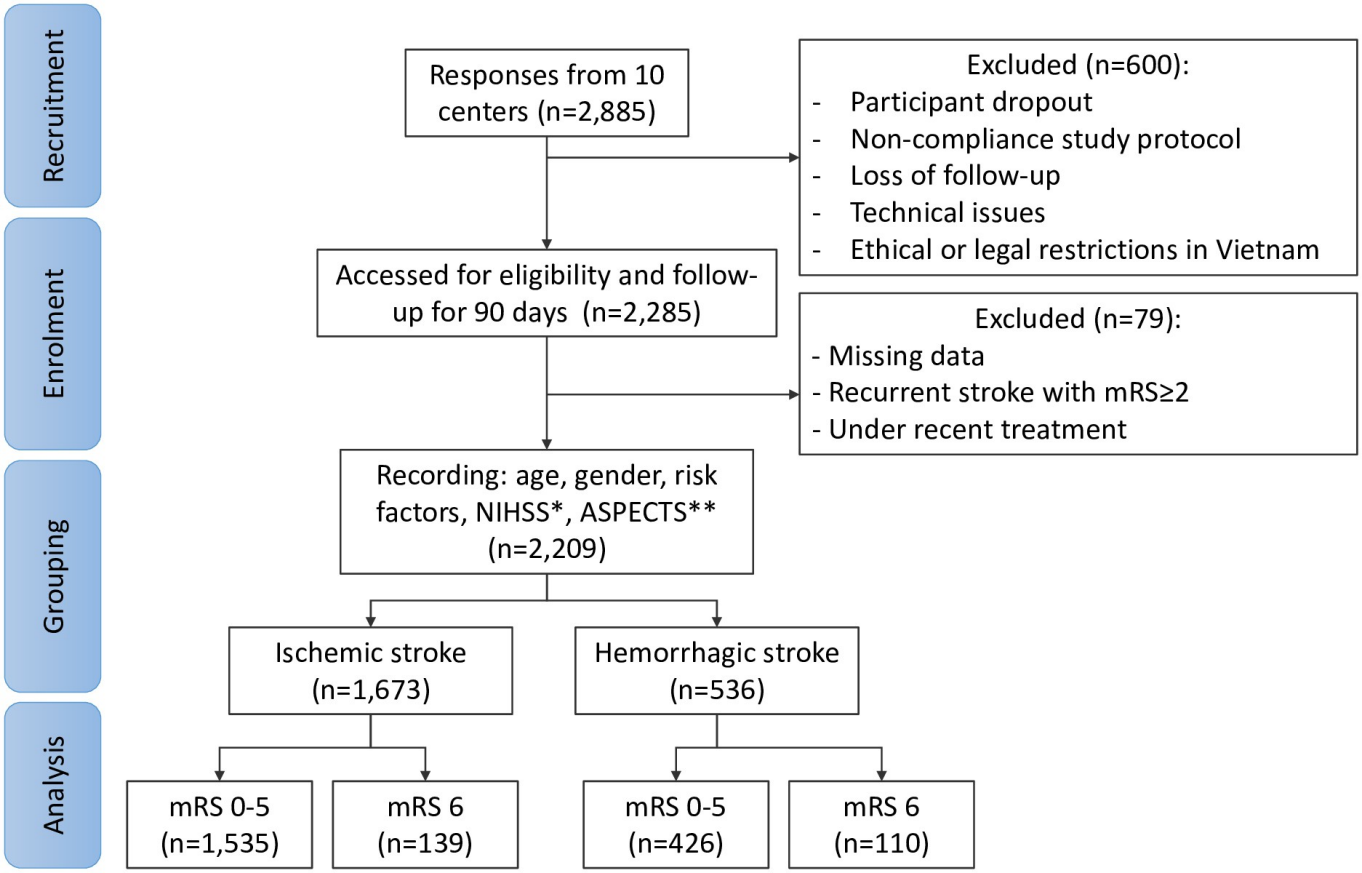
CONSORT diagram of the inclusion and exclusion subjects in this research. *: NIHSS (n = 2203) is the National Institutes of Health Stroke Scale, **: ASPECTS (n = 723) is the Alberta Stroke Program Early CT-Scan.

All study participation was described as voluntary. As the study carried little to no risk to the patient, there was a waiver of informed consent (approved by the ethics committee of Bach Mai Hospital). Patients were freely withdrawn from the study at any time. Data reporting followed the Strengthening the Reporting of Observational Studies in Epidemiology (STROBE) guidelines. The research has been performed following the Declaration of Helsinki. Further information on the data collection can be found in Fig 1. Patients were included if they were diagnosed with stroke and were seeking help at a participating health centre within 3 days from symptom onset. The excluded criteria were participants who lacked complete 90-day post-treatment data as per the medical record, experienced a recurrent stroke with a mRS score of 2 or above, or had already received treatment from a referring hospital.

Acute ischemic stroke or intracerebral hemorrhage patients were selected based on an updated definition of stroke [5]. Ischemic stroke was defined as an episode of neurological impairment brought on by a focal infarction of the brain, spinal cord, or retina. The term "intracerebral hemorrhage" refers to the quickly emerging clinical symptoms of neurological dysfunction that are linked to a non-traumatic focused collection of blood within the ventricular system or brain parenchyma. Subarachnoid hemorrhage was characterized as quickly emerging symptoms or indicators of headache and/or neurological dysfunction resulting from bleeding into the subarachnoid space (the area between the brain’s pia mater and arachnoid membrane), which was not caused by trauma. Certified stroke neurologists assessed the baseline National Institutes of Health Stroke Scale (NIHSS) scores of each participant. Etiologically, stroke subtypes were identified using the Trial of Organization 10172 in Acute Stroke Treatment categorization (TOAST) [6]. Finally, functional impairment was prospectively evaluated at discharge, 30 days, and 90 days post-treatment according to the modified Rankin Scale (mRS).

During hospitalization, we collected the following information: age, gender, medical history (hypertension, diabetes mellitus, smoking, atrial fibrillation), NIHSS, ASPECTS (Alberta Stroke Program Early CT-Scan), and TOAST classification. For intracerebral hemorrhage (ICH) patients, the variables reviewed included age, gender, medical history, NIHSS at admission, and hemorrhage location. The primary outcome of the study was mortality or mRS 6 at 90 days post-treatment.

### Posthoc and statistical analysis

The independent variable was mRS at discharge 90 days post-treatment, which was dichotomized into survival (mRS 0–5) and death (mRS 6). Patients who did not have data of mRS at day 90 post-treatment were considered as missing values. Missing data were excluded before data analysis. For comparing categorical variables, either Fisher’s exact test or Pearson’s chi-square test was employed appropriately. The Independent variable was based on the study hypothesis and previous studies, independent variables were chosen. We employed both univariable and multivariable logistic regression to assess the relationship between these independent variables and the specified dependent variable. The variable with a p-value less than 0.05 in the univariable analysis was considered statistically significant and included in the multivariable model.

The odds ratios (ORs) for the comparison of the two groups were reported with 95% confidence intervals (CIs). The ORs were adjusted for factors that could affect the outcome at discharge 90 days post-treatment in ischemic stroke and hemorrhagic stroke, including age, gender, hypertension, diabetes, smoking, atrial fibrillation) risk factors and NIHSS. The ORs were reported with 95% confidence intervals (CIs). A p-value of less than 0.05 was deemed statistically significant. Data analysis was performed using SPSS software version 26 (IBM Co., Armonk, NY, USA). Subgroup characteristics were summarized using descriptive statistics.

### Preliminary explainable machine learning model

Off the shelf explainable machine learning (XML) model aimed at identifying mortality risk after 90 days of stroke onset using XGBoost (https://xgboost.readthedocs.io/) and SHAP (https://shap.readthedocs.io/) [7]. This is the preliminary work in utilizing machine learning in comparison with traditional stroke scores in predicting mortality after 90-day stroke treatments. Firstly, a comprehensive dataset comprising demographic information, clinical variables, and imaging findings from a multicenter cohort of Vietnamese stroke patients was pre-scanned and pre-processed to exclude missing data, normalizing features, and encoding categorical variables. To develop the ML model, we implement interpretable algorithms, extreme gradient boosting, and XGBoost. Model performance evaluated using metrics such as accuracy, precision, sensitivity, specificity, and f1 score. Additionally, to enhance model transparency and interpretability, we employ SHAP (SHapley Additive exPlanations) values to elucidate the impact of individual features on the model’s predictions. All parameters for the model are included in S1 Appendix. Finally, the developed model undergoes rigorous validation, training 80% to leave one out, and 20% for validation. Results be shown in the test set only.

## Results

**Fig 2:**
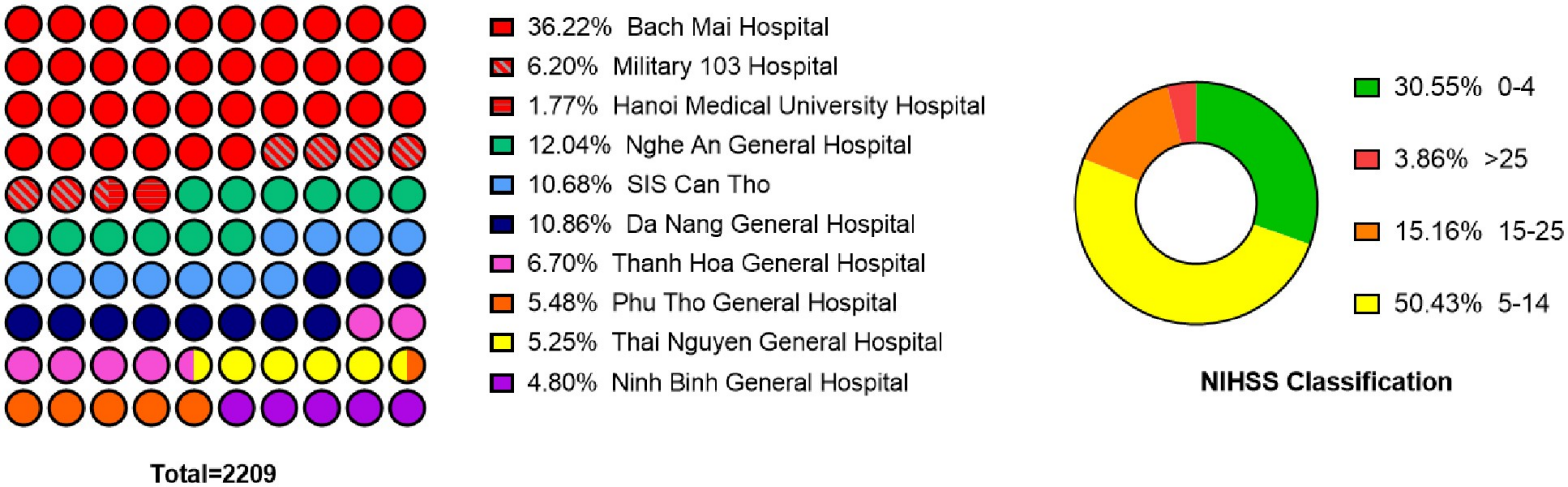
Percentage plots of the data distribution and the percentage of the NIHSS classification from 10 stroke centres/units/departments across Vietnam.

Upon completion of data collection, 2,285 patients were identified who met the inclusion criteria outlined in the study. 2209 patients were taken into analysis with 1673 ischemic strokes and 536 hemorrhage stroke subjects. Table 1 summarises the clinical characteristics of the study population. The mean age was 65.4 ± 13.7 years, with 1032 (45.7%) aged below 65 years. Men constituted 1357 participants, comprising 61.4% of the cohort. According to Fig 2, among ten stroke centres, the Stroke Center of Bach Mai Hospital was the primary contributor, enrolling 800 patients (36.2%), followed by Nghe An General Hospital from Vietnam’s North Central Region, with 266 patients (12.0%). Institutions from the Central and Southern regions of Vietnam cumulatively enrolled 624 patients, representing 28.3% of the total study population. Ischemic stroke was the predominant subtype, affecting 75.7% of the cohort, followed by intracerebral hemorrhage at 19.9%. In the entire cohort, hypertension was present in 77.2%, diabetes in 17.7%, smoking in 15.0% and atrial fibrillation in 5.6%. The median (IQR) NIHSS was 8 (4–12). The mortality for ischemic stroke and intracerebral hemorrhage were 8.2% and 20.5%, respectively.

**Table 1:** Overall summary of the clinical characteristics in ischemic and intracerebral hemorrhage patients (n = 2209).

Characteristics	Count (percentage)
**Age (65.4 ± 13.7)**	
**≤65 years old**	1032 (46.7)
**Gender (Male)**	1357 (61.4)
**Risk factors**	
**Hypertension**	1705 (77.2)
**Diabetes**	391 (17.7)
**Smoking**	331 (15.0)
**Atrial fibrillation**	124 (5.6)
**Stroke classification**	
**Ischemic stroke**	1673 (75.7)
**Intracerebral hemorrhage**	439 (19.9)
**SAH**	97 (4.4)
**Mortality**	248 (11.2)
**Ischemic stroke (n = 1673)**	138 (8.2)
**Hemorrhagic stroke (n = 536)**	110 (20.5)
**NIHSS**	8 (4–12)*

SAH: subarachnoid hemorrhage; NIHSS: National Institutes of Health Stroke Scale.

* median (IQR) was shown.

The clinical characteristics of ischemic stroke, categorized by mRS scores of 6 and 0–5 are presented in Table 2. The prevalence of atrial fibrillation was far higher among fatal patients compared to those who survived (17.4% vs. 6.4%, p<0.001). Both higher baseline NIHSS (p < 0.001) and lower baseline ASPECTS (p = 0.001) were associated with increased mortality at day 90. Following the TOAST classification, there were elevated rates of large artery atherosclerosis (40.6% vs. 23.8%, p < 0.001) and cardioembolic etiology (19.6% vs. 7.7%, p<0.001) in patients who had succumbed, while small vessel disease was high prevalent in the deceased cohort (22.5 vs. 44.0%, p < 0.001).

**Table 2:** Comparison of multiple factors between mRS 6 and mRS 0–5 in patients with ischemic stroke (n = 1673). Fisher’s exact test was performed.

Factors	mRS 6	mRS 0–5	p-value
**Age > 65-year-old**	99 (71.7)	880 (57.3)	0.001
**Gender: Male**	85 (61.6)	930 (60.6)	0.82
**Risk factors**			
**Hypertension**	99 (71.7)	1204 (78.4)	0.07
**Diabetes**	33 (23.9)	301 (19.6)	0.23
**Smoking**	19 (13.8)	222 (14.5)	0.82
**Atrial fibrillation**	24 (17.4)	98 (6.4)	<0.001
**NIHSS >14**	68 (49.3)	153 (10.0)	<0.001
**TOAST classification**			
**Small vessel disease**	31 (22.5)	676 (44.0)	<0.001
**Large artery atherosclerosis**	56 (40.6)	366 (23.8)	<0.001
**Cardio-embolism**	27 (19.6)	118 (7.7)	<0.001
**Other determined etiology**	2 (1.4)	15 (1.0)	0.65
**Undetermined etiology**	22 (15.9)	360 (23.5)	0.04

NIHSS: National Institutes of Health Stroke Scale; TOAST: **Trial of ORG 10172 in acute stroke treatment**.

In patients with intracerebral hemorrhage, the clinical attributes are shown based on mRS scores of 0–5 and 6 and delineated in Table 3. A significant association was observed between higher baseline NIHSS and 90-day mortality (p < 0.05). In addition, the presence of subarachnoid hemorrhage (36.4% vs. 23.0%, p = 0.005) and intraventricular hemorrhage (54.2% vs. 26.6%, p <0.001) were associated with a higher rate of 90-day mortality. In contrast, intraparenchymal hemorrhage (80.4% vs. 77.5%, p = 0.52) had no statistical significance with rates of 90-day mortality.

**Table 3:** Comparison of multiple factors between mRS 6 and mRS 0–5 in patients with hemorrhage stroke (n = 536). Fisher’s exact test was performed.

Factors	mRS 6	mRS 0–5	p-value
**Age**			
**> 65-year-old**	37 (33.6)	161 (37.8)	0.42
**Gender**			
**Male**	74 (67.3)	268 (62.9)	0.34
**Risk factors**			
**Hypertension**	75 (68.2)	327 (76.8)	0.06
**Diabetes**	8 (7.3)	49 (11.5)	0.20
**Smoking**	17 (15.5)	73 (17.1)	0.67
**Atrial fibrillation**	1 (0.9)	1 (0.2)	0.37
**NIHSS >14**	90 (81.8)	108 (25.5)	<0.001
**Intracerebral hemorrhage**			
**Intraparenchymal hemorrhage**	86 (80.4)	320 (77.5)	0.52
**Intraventricular hemorrhage**	58 (54.2)	110 (26.6)	<0.001
**Subarachnoid hemorrhage**	39 (36.4)	95 (23.0)	0.005

NIHSS: National Institutes of Health Stroke Scale; TOAST: **Trial of ORG 10172 in acute stroke treatment**.

**Fig 3:**
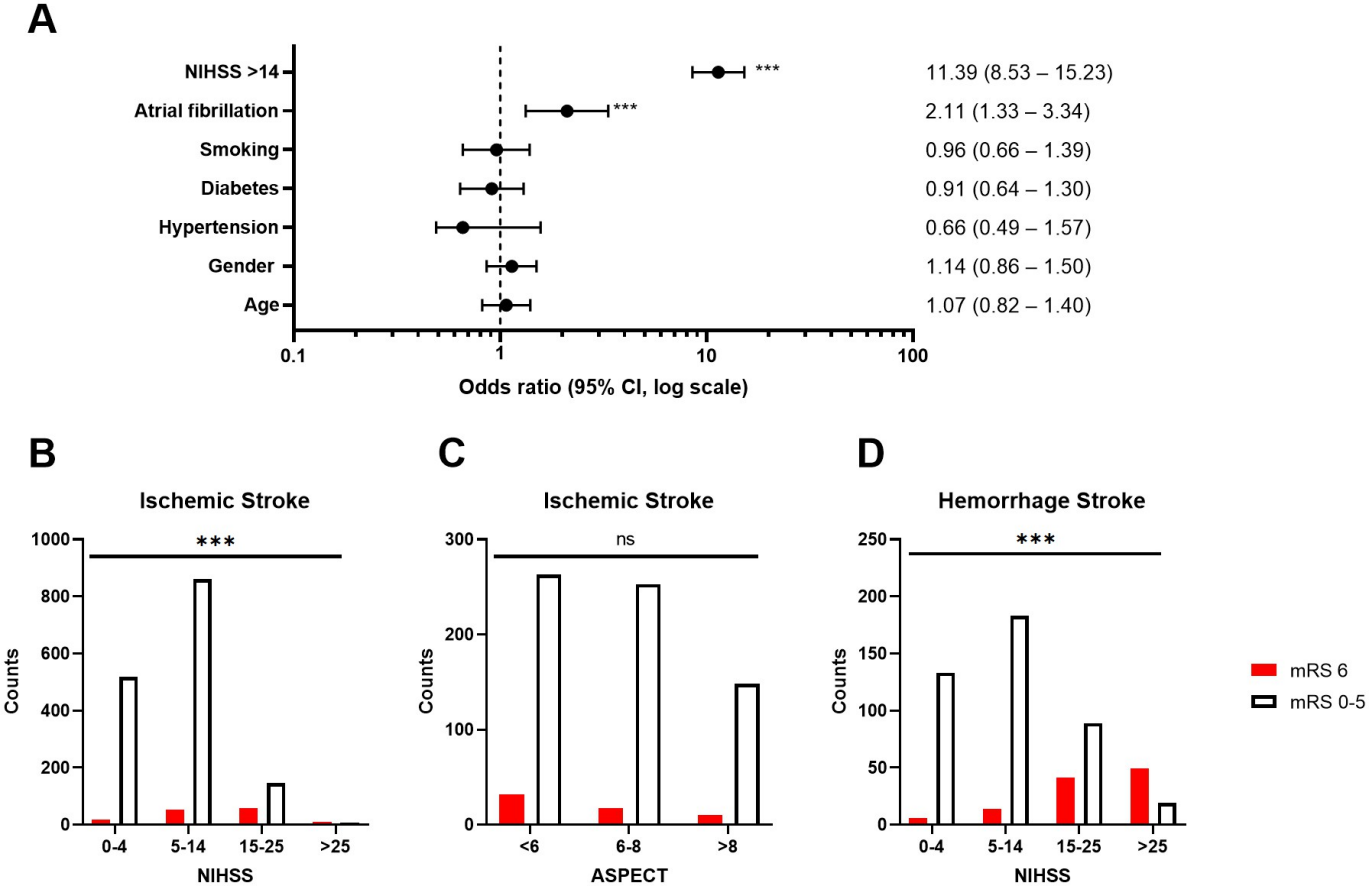
Factors associated with 90 days mortality in ischemic and hemorrhage stroke patients. Panel A shows the Odds ratio plot for the factors affecting Mortality 90 Days after treatment in all patients. Panels B and C show the patients with Ischemic Stroke in NIHSS and ASPECT scores. Panel D shows the patients with Hemorrhage Stroke in NIHSS score. The Pearson’s chi-square test was performed with ns as non-significant, and ***: p<0.001.

The variables that impacted mortality in both groups of ischemic stroke and intracerebral hemorrhage 90 days post-treatment were demonstrated in Fig 3. Accordingly, in both groups of patients with ischemic or hemorrhagic stroke, mortality at day 90 after treatment was influenced by the NIHSS scale, atrial fibrillation, hypertension, and recurrent stroke. NIHSS > 14 is a variable that impacted mortality in both groups of patients with ischemic stroke and intracerebral hemorrhage 90 days post-treatment (OR, 11.39; 95% CI, 8.53–15.23). There is no statistical significance in mortality at 90 days after treatment in patients with hypertension compared to patients without hypertension (OR, 0.66; 95%CI, 0.49–1.57). Patients who had atrial fibrillation show a statistical association of mortality at 90 days after treatment compared to patients with no report of atrial fibrillation (OR, 2.11; 95%CI, 1.33–3.34).

Table 4 shows the factors associated with mortality after 90 days of treatment in ischemic stroke patients using the univariable and multivariable logistic regression models. Patients aged ≥ 65 years exhibited an increased risk of mortality (OR 1.89; 95% CI, 1.29–2.77). Patients with a history of atrial fibrillation faced a 3.09-fold higher risk of mortality at 90 days (OR 3.09; 95% CI 1.90–5.02). There were higher odds of mortality with increasing baseline NIHSS scores (NIHSS >14: OR 8.75, 95% CI 6.02–12.71). Lower strata of ASPECTS were also associated with greater odds of mortality (ASPECTS <6: OR 1.81, 95% CI 1.06–3.09). Furthermore, the presence of large artery atherosclerosis (OR 2.18, 95% CI 1.52–3.13) and cardio embolism (OR 2.92, 95% CI 1.84–4.63) were identified as additional factors impacting 90-day mortality in patients with ischemic stroke. Table 5 reveals the results of the univariable logistic regression models to evaluate the factors affecting mortality after 90 days of treatment in patients with intracerebral hemorrhage. In which factors including NIHSS > 14 (OR, 13.17; 95% CI, 7.74–22.40, subarachnoid hemorrhage (OR, 1.92; 95% CI, 1.223.02), intraventricular hemorrhage (OR, 3.26; 95% CI, 2.10–5.06–2.13) were factors affecting mortality after 90 days of treatment.

**Table 4:** Odd ratio results of the factors associated with mortality 90 days after treatment in ischemic stroke (n = 1673).

Factors	OR (CI 95%)
**Gender (Male)**	1.04 (0.73–1.49)
**Age, years>65**	1.89 (1.29–2.77)
**Risk factors**	
**Hypertension (Yes)**	0.70 (0.47–1.03)
**Smoking (Yes)**	0.94 (0.57–1.56)
**Atrial fibrillation (Yes)**	3.09 (1.90–5.02)
**Diabetes (Yes)**	1.29 (0.85–1.94)
**NIHSS classification >14**	8.75 (6.02–12.71)
**ASPECTS <6**	1.81 (1.06–3.09)
**TOAST classification**	
**Small vessel occlusion (Yes/No)**	0.37 (0.24–0.56)
**Large artery atherosclerosis (Yes/No)**	2.18 (1.52–3.13)
**Cardioembolism (Yes/No)**	2.92 (1.84–4.63)
**Other determined etiology (Yes/No)**	1.49 (0.34–6.58)
**Undetermined etiology (Yes/No)**	0.62 (0.39–0.99)

NIHSS: National Institutes of Health Stroke Scale. ASPECTS: Alberta Stroke Program Early CT–scan. TOAST: **Trial of ORG 10172 in acute stroke treatment**.

**Table 5:** Odd ratio results of the factors associated with mortality 90 days after treatment in intracerebral hemorrhage (n = 536).

Factors	OR (95% CI)
**Age>65**	0.83 (0.54–1.30)
**Gender (Male/Female)**	1.21 (0.78–1.89)
**Risk factors**	
**Hypertension (Yes/No)**	0.65 (0.41–1.03)
**Diabetes (Yes/No)**	0.60 (0.28–1.32)
**Smoking (Yes/No)**	0.88 (0.50–1.57)
**Atrial fibrillation**	3.90 (0.24–62.84)
**NIHSS >14**	13.17 (7.74–22.40)
**Location of hemorrhage**	
**Intraparenchymal hemorrhage (Yes/No)**	1.19 (0.70–2.02)
**Intraventricular hemorrhage (Yes/No)**	3.26 (2.10–5.06)
**Subarachnoid hemorrhage (Yes/No)**	1.92 (1.22–3.02)

NIHSS, National Institutes of Health Stroke Scale.

**Fig 4:**
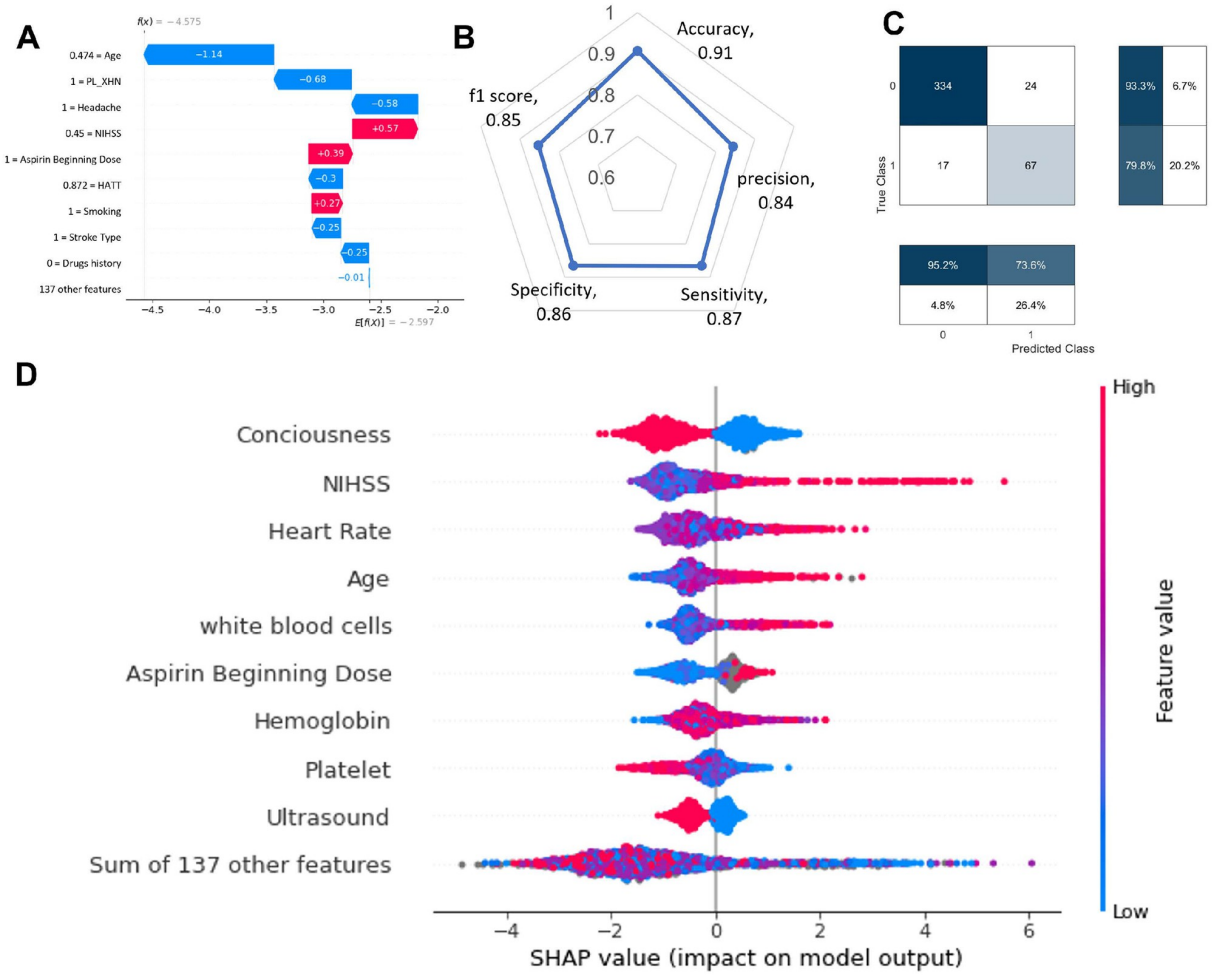
Explainable machine learning outcome 90 days mortality prediction and associated risk factors. Panel A shows the example of the explainable machine learning model predicting the mortality rate of an individual subject. Panel B is the star plot of sensitivity, specificity, precision, accuracy and f1-score of the machine learning model. Panel C shows the confusion matrix of the model in the prediction of death (1 death, 0-alive) after 90 days. Panel D shows the ranking of importance of SHAP values in the model prediction of death.

Fig 4 shows the preliminary performance of the XML in the prediction of stroke deaths after 90 days of discharge. Panel A illustrates an example of the different risk factors that impact the decision of the model in one single subject. Age and NIHSS are the top priority. Panels B and C show the quantitative of the machine learning performance in the prediction of 90-day mortality with 0.91 accuracy and f1 = 0.85. Panel D shows SHAP value from high to low ranking importance to the rate of death after 90 days post-stroke discharge. Interestingly consciousness level got the highest ranking, followed by NIHSS number, heart rate and age.

## Discussion

This prospective study showed the mortality in patients with ischemic stroke and hemorrhagic stroke was 8.2% and 20.5%, respectively. These results align with previous studies conducted in Southeast Asian countries [8, 9]. In ischemic stroke, patients with higher baseline NIHSS, cardioembolic stroke etiology, atrial fibrillation, and large artery atherosclerosis had a higher likelihood of 90-day mortality. Earlier data in the Vietnamese population have demonstrated an association between atrial fibrillation and cardiac thrombosis and an elevated risk of mortality among stroke patients [10]. Notably, the utilization of anticoagulation therapy when indicated remains relatively low in Vietnam. Explainable machine learning could be a potential support tool for stroke prediction and support stroke care in low and low-middle-income countries.

In patients with ischemic stroke, the NIHSS score was significantly associated with 90-day mortality with an OR of 11.39 [11], and as expected, patients with lower ASPECTS also had higher odds of 90-day mortality. In patients with cardiogenic stroke, it is thought that patients often develop severe neurological symptoms, early mortality, and poor outcomes [12]. The prognosis of patients with a large vessel occlusion remains poor [13] with 70–80% mortality [14], life-threatening brain oedema causing transtentorial herniation is known as the most common cause of death. Additionally, compared to patients who had less severe strokes, patients with large vessel occlusion typically require a longer hospital stay, which is partially explained by the higher complication rates, of which, 3% of patients died during hospitalization [15]. Ischemic stroke patients with cardio-embolism and large artery atherosclerotic stroke experienced significantly higher in-hospital mortality than those without [16].

In the total involving 2,885 patients, data for 600 patients (20.8%) was excluded. This missing data could be attributed to several factors, including participant dropout, non-compliance with the study protocol, and loss of follow-up. Managing the data collection in 10 units led to technical issues with data collection infrastructure, instruments, data entry errors, and logistical constraints. Additionally, due to ethical or legal restrictions in Vietnam, patient reluctance to provide certain information, and administrative issues further contribute to missing data. Due to the complexity of the healthcare system in Vietnam for stroke treatment, patients may be transferred to a second hospital for advanced care. To make the analysis more appropriate, an additional 76 patients were excluded from the analysis due to missing data, recurrent strokes, and ongoing treatment from previous hospitals or healthcare units. Addressing these challenges requires careful planning, robust data management practices, and appropriate statistical methods to maintain the study’s integrity and validity in the future.

According to our preliminary results, there are correlation between explainable machine learning in the prediction of mortality with standard classification using stroke scores. The factors that influence mortality at 90 days post-treatment in stroke patients with intracranial hemorrhage in this study included hypertension, baseline NIHSS score, and the presence of intraventricular hemorrhage. NIHSS and age are correlated between the machine learning model and clinical statistic results. Furthermore, high blood pressure accounted for 77.2 of stroke patients which is very high compared to other studies in the same settings [17]. The machine learning model picked up the symptoms, such as high values of heart rate low number of white blood cells, and haemoglobin (Fig 4D). The importance of blood pressure management and the close relationship between blood pressure and hemorrhagic stroke, have been supported by earlier investigations [18, 19]. There are many interesting features to compare explainable machine learning with statistical values. The machine learning model might be able to pick up sensitive values, however, robustness to noise might be a limitation for these high-dimensional models.

In addition, in patients with intracerebral hemorrhage, blood pressure control plays an important role, especially in the acute stage which may have a significant impact on the patient’s prognosis [20]. However, in our study, there was no statistical significance between blood pressure and mortality 90 days post-treatment. We assumed that can be the result of many deaths recorded at an earlier stage in patients with hypertension. Patients with intraventricular hemorrhage had mortality at day 90 which was three-fold higher than patients who had no bleeding at this site (95% CI; 2.10–5.06, p <0.001). This has also been observed in previous studies, with a mortality rate of up to 50% in patients with intraventricular hemorrhage [21] since this causes acute ventricular dilatation and increased intracranial pressure which leads to brain herniation [22]. Understanding factors that are associated with mortality after 90 days can inform stroke neurologists and neurosurgeons in guiding prognosis, potential treatment interventions, and discussion with family members.

Although there are some strengths to our study, our report contains several limitations. The time intervals in treating acute stroke patients as well as the length of hospital stay according to previous studies are factors that affect treatment results after 90 days [23], however, these factors were not accounted for in the study. In addition, we collected data on factors that occur during hospital treatment. All data was gathered in a general stroke care setting with 2885 participants in 10 stroke centres throughout Vietnam. 20,8% of the collected data was excluded due to the data collection. Rehabilitation and recovery factors that occur when the patient is discharged from the hospital might also affect mortality outcomes [24]. Although a machine learning model was developed to demonstrate the agreement between machine learning and traditional classification using stroke scores (mRS, NIHSS score, and ASPECT score), more sophisticated analysis could be carried on in the future analysis, such as comparing ischemic or haemorrhagic stroke, and analysis according to sex. A follow-up study directly comparing machine learning with conventional medical statistical methods could be a valuable area of exploration.

## Conclusion

This multicentre study was conducted at ten stroke facilities in Vietnam to examine clinical factors that were associated with 90-day mortality. Notably, cardiogenic embolism, ASPECTS, and NIHSS scores emerged as variables significantly linked to mortality in patients with ischemic stroke. In the case of individuals suffering from intracerebral hemorrhage, NIHSS score, and the presence of intraventricular hemorrhage were associated with mortality. An explainable machine learning model could be a potential support to predict mortality risk post-stroke after 90 days in developing countries, especially Vietnam.

## Supporting information

S1 Appendix Parameter for the machine learning algorithm. (DOCX)
