# Correction: Factors associated with 90-day mortality in Vietnamese stroke patients: Prospective findings compared with explainable machine learning, multicenter study

**DOI:** 10.1371/journal.pone.0343862

**Published:** 2026-02-26

**Authors:** Ton Duy Mai, Dung Tien Nguyen, Cuong Chi Tran, Hai Quang Duong, Hoa Ngoc Nguyen, Duc Phuc Dang, Hai Bui Hoang, Hong-Khoi Vo, Tho Quang Pham, Hoa Thi Truong, Minh Cong Tran, Phuong Viet Dao

The last two authors, Minh Cong Tran and Phuong Viet Dao, should not have been attributed equal contribution to this work. There is also an error in affiliation 1. The correct affiliation 1 is: Faculty of Stroke and Cerebrovascular Disease, VNU-University of Medicine and Pharmacy, Ha Noi, Viet Nam.
