# Supplementary material for: Factors associated with 90-day mortality in Vietnamese stroke patients: Prospective findings compared with explainable machine learning, multicenter study
Source: PLoS One. 2024 Sep 20;19(9):e0310522. doi: 10.1371/journal.pone.0310522 (PMC11414902; doi:10.1371/journal.pone.0310522)
Supplement: S1 Appendix — (DOCX) [file pone.0310522.s001.docx]

**Appendix Table 1: Parameter for the machine learning algorithm**

| **Number** | **Parameters** |
| --- | --- |
| 1 | Age |
| 2 | Gender |
| 3 | TIA history |
| 4 | Smoking history |
| 5 | Atrial fibrillation history |
| 6 | Heart failure, reflux failure |
| 7 | Mechanical heart valve |
| 8 | Biological heart valve |
| 9 | Previous MI |
| 10 | Angina pectoris |
| 11 | Pacemaker |
| 12 | PCI bypass |
| 13 | Previous stroke history |
| 14 | Previous TIA |
| 15 | Current medications |
| 16 | LDL/HDL cholesterol |
| 17 | Coronary artery stenosis >50% |
| 18 | Coronary artery dissection, coronary stent |
| 19 | PAD history |
| 20 | Renal failure history |
| 21 | Diabetes history |
| 22 | Cancer history |
| 23 | Dementia history |
| 24 | APS history |
| 25 | Gastric ulcer history |
| 26 | Substance abuse history |
| 27 | Time to stroke |
| 28 | Heart rate |
| 29 | Temperature |
| 30 | SBP |
| 31 | DBP |
| 32 | WBC |
| 33 | Hemoglobin |
| 34 | Platelets |
| 35 | Consciousness |
| 36 | Speech disturbances |
| 37 | Motor paralysis |
| 38 | Sensory disturbances |
| 39 | Dizziness face |
| 40 | Visual disturbances |
| 41 | Confusion |
| 42 | Headache |
| 43 | NIHSS |
| 44 | CT scan |
| 45 | CTA scan |
| 46 | MRI scan |
| 47 | Doppler transverse axonal scan |
| 48 | CTA MRA |
| 49 | Coronary ultrasound |
| 50 | DSA |
| 51 | Thoracic echocardiography |
| 52 | Esophageal echocardiography |
| 53 | Electrocardiogram |
| 54 | Holter electrocardiogram |
| 55 | Vascular hemorrhage |
| 56 | Strangulated hemorrhage |
| 57 | Subarachnoid hemorrhage |
| 58 | CVT |
| 59 | Cerebral infarction |
| 60 | Vascular stenosis, endoscopic > 50% |
| 61 | Sacroiliac stenosis, pleural lesion |
| 62 | Cardiovascular disease |
| 63 | Cardiac thrombus |
| 64 | Heart failure EF < 30% |
| 65 | Patent foramen ovale PFO |
| 66 | Atrial fibrillation detected |
| 67 | Initial aspirin |
| 68 | Initial dose aspirin |
| 69 | 48-hour aspirin |
| 70 | 48-hour aspirin |
| 71 | Withdrawal aspirin |
| 72 | Withdrawal dose aspirin |
| 73 | Initial clopidogrel |
| 74 | Clopidogrel 48 hours |
| 75 | Clopidogrel withdrawal |
| 76 | VKA initial |
| 77 | VKA 48 hours |
| 78 | VKA discharge |
| 79 | Rivaroxaban initial |
| 80 | Rivaroxaban 48 hours |
| 81 | Rivaroxaban discharge |
| 82 | Dabigatran initial |
| 83 | Dabigatran 48 hours |
| 84 | Dabigatran discharge |
| 85 | Apixaban initial |
| 86 | Apixaban 48 hours |
| 87 | Apixaban discharge |
| 88 | Beta blockers initial |
| 89 | Beta blockers 48 hours |
| 90 | Beta blockers discharge |
| 91 | Diuretics initial |
| 92 | Diuretics 48 hours |
| 93 | Diuretics |
| 94 | ACEI initial |
| 95 | ACEI 48 hours |
| 96 | ACEI discharge |
| 97 | ARB initial |
| 98 | ARB 48 hours |
| 99 | ARB discharge |
| 100 | CCB initial |
| 101 | CCB 48 hours |
| 102 | CCB discharge |
| 103 | Statin initial |
| 104 | Statin 48 hours |
| 105 | Statin discharge |
| 106 | Diabetes medications initial initial |
| 107 | Diabetes medication 48 hours |
| 108 | Diabetes medication withdrawal |
| 109 | Initial insulin |
| 110 | Insulin 48 hours |
| 111 | Discharge insulin |
| 112 | Thrombolytic |
| 113 | Thrombolysis |
| 114 | Stroke classification |
| 115 | Cerebral infarction etiology |
| 116 | Hyperlipidemia history classification |
| 117 | Age classification |
| 118 | filter_$ |
| 119 | Supratentorial cerebral hemorrhage classification |
| 120 | Stroke classification |
| 121 | Cerebral hemorrhage classification |
| 122 | Subarachnoid hemorrhage classification |
| 123 | CVT classification |
| 124 | Under 4.5 hours |
| 125 | From 4.5 to 6 hours |
| 126 | From 6 to 04 hours |
| 127 | After 04 hours |
| 128 | Small vessel etiology |
| 129 | Large vessel etiology |
| 130 | Cardiac etiology |
| 131 | Other etiology |
| 132 | Age classification under 50 years |
| 133 | Age classification under 60 years |
| 134 | Age classification under 70 years |
| 135 | Age classification under 55 years |
| 136 | Age classification under 65 years |
| 137 | Unknown etiology |
| 138 | Gender Female |
| 139 | Age classification over 75 years |
| 140 | Age over 70 |
| 141 | Age classification over 65 |
| 142 | Age classification over 80 |
| 143 | Age classification over 50 |
| 144 | Age classification over 45 |
| 145 | Age classification over 55 |
| 146 | Age classification over60 |
